# Ocular and General Proprioception in Dyslexic Children: A Review of Their Diurnal and Nocturnal Dysfunctions and Their Repercussions

**DOI:** 10.3390/vision9020044

**Published:** 2025-05-20

**Authors:** Patrick Quercia, Kalvin Chavet, Jérémie Gaveau

**Affiliations:** 1 INSERM UMR1093-CAPS, UFR des Sciences du Sport, Université Bourgogne Franche-Comté, F-21000 Dijon, France; jeremie.gaveau@u-bourgogne.fr; 2 UFR des Sciences du Sport, Université de Bourgogne, F-21000 Dijon, France; kalvinchavet4@gmail.com

**Keywords:** dyslexia, ocular proprioception, spatial localization, child sleep, UARS, posture, balance, sensorimotor plasticity, multisensory integration

## Abstract

We provide a summary of the research conducted in our laboratory on the relationship between ocular proprioception, general proprioception, and dyslexia. Dyslexic children show a marked proprioceptive deficit which affects motor control, attention and spatial perception. The spatial disturbances are expressed by the presence of a vertical microheterophoria which has very specific characteristics. It is associated with abnormal tone of the oblique muscles and can be modified by means of very low powered prisms and/or remote sensory stimulation. When ocular proprioception is modified, sounds cause stochastic visual losses. This may interfere with the association between phonemes and graphemes, which is necessary for learning to read. The effects of a generalized nocturnal proprioceptive disorder may play a role in the abnormal brain development that has been observed in dyslexic children.

## 1. Introduction

Dyslexia affects around 10% of schoolchildren, which means that an ophthalmologist is likely to encounter at least one dyslexic patient on a daily basis, often without being aware of it. The interaction between ocular proprioception, general proprioception and dyslexic disorders is a relevant line of research that could contribute to better care for these children.

Dyslexia is defined as a long-lasting difficulty in learning to read, occurring despite normal intelligence, the absence of proven sensory or neurological deficits, and a suitable school and socio-cultural environment. Reading delay is resistant to interventions (pedagogical, re-educational).

Its cause remains a mystery, although the prevailing hypothesis is that a genetic deficit is responsible for a neurodevelopmental disorder with negative consequences for phonological awareness, i.e., the ability to perceive, manipulate and represent the sounds of language [1]. This phonological deficit disrupts the association between sounds (phonemes) and letters or groups of letters (graphemes). This theory considers the phonological disorder to be causal, disconnected from any sensorimotor contingency. The auditory and visual sensory peculiarities accompanying the reading disorder are considered comorbidities [2,3]. Numerous studies have also noted the existence of motor difficulties in dyslexic children [4].

This article presents the results of over 20 years of research into proprioception in dyslexic children at INSERM Unit 1093 Cognition, Action et Plasticité Sensorimotrice at the Université Bourgogne Europe. These results highlight neurophysiological implications and innovative therapeutic perspectives.

The guiding thread of this work is based on an approach that favors a global perspective on the interactions between perception, action, and cognition and consequently considers motor disorders as a key factor in the possible onset of cognitive development disorders. It has been guided by 5 key principles:

(1) Integrative approach to theories: No theory that has been proposed to explain dyslexia, whether sensory (magnocellular, visual, etc.), motor (cerebellar) or cognitive (phonological, visual-attentional, etc.), is favored or discarded, as each is based on solid arguments,
(2) Embodied cognition: Brain function is considered in interaction with the body and the environment, avoiding a strictly disconnected approach to both exteroceptive and interoceptive sensory data,
(3) Reconsidering comorbidities: Rather than seeing them as independent disorders, they could be manifestations of the same neurodevelopmental dysfunction, suggesting a common origin,
(4) Neurogenesis and the mechanisms of cerebral plasticity present particular temporal dynamics. It is therefore crucial not only to observe what dyslexics were like before learning to read, but also to follow them over the entire nycthemeris in order to analyze the evolution of these processes throughout the sleep-wake cycle,
(5) Back to medical basics: Clinical observation and a holistic approach to the patient are essential. This approach naturally led to an interest in the work of the Portuguese physician Henrique Martins da Cunha, who associated dyslexia with a proprioceptive and visual dysfunction, responsible for disparate bodily and cognitive signs that appear unrelated to those unfamiliar with the physiology of proprioception [5].

## 2. Proprioceptive Dysfunction and Dyslexia

Proprioception, the interoceptive sense, is an essential component of sensorimotor control. It relies on mechanoreceptors located in muscles, tendons, ligaments and joints. Its operation is closely linked to the efference copy sent to the brain structures involved in sensorimotor perception and coordination during a motor command.

**Figure 1:**
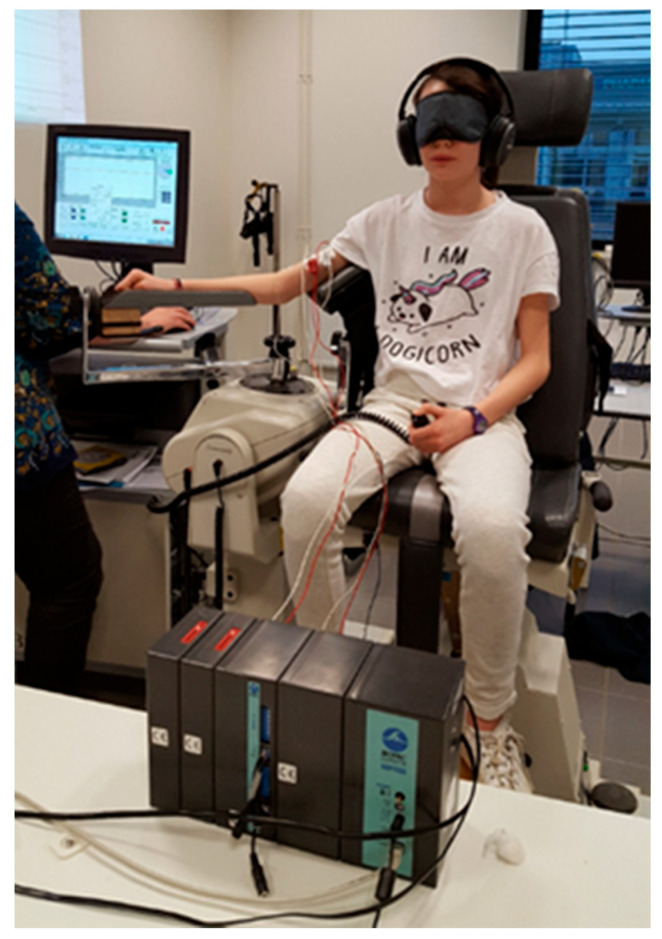
Repeated measurement of arm proprioceptive acuity using a digital ergometer in the laboratory. The child is isolated from all sound and visual stimulation, and the absence of voluntary arm movement is monitored by surface EMG. The child is asked to signal (by pressing the button on his left hand) when he/she detects the arm movement created by the device.

Proprioceptive acuity can, however, be measured without being influenced by the efference copy by evaluating the detection time of a strictly passive movement, created by a digital ergometer associated with a surface EMG monitoring the absence of voluntary contraction (Figure 1).

This measurement, applied to dyslexic children, shows that at the slowest speed (0.25°·s^−1^), during passive elbow movements, the reaction time, considered to reflect the child’s proprioceptive acuity, is twice as long and more variable than in controls [6]. There is a strong correlation between the proprioceptive acuity index and the reading ability index. The former was calculated as the normalized mean of the average and variable reaction times at the slowest speed. The latter corresponded to the normalized mean of the four scores traditionally used to assess dyslexia in France (reading speed index and accuracy index in the Alouette test, Timé 3 score, and NEPSY2 result). Proprioceptive acuity is also significantly correlated with phonological abilities (Pearson R = 0.45, *p* = 0.008. This correlation is not the result of a bimodal distribution of data between dyslexics and controls. When grouped together, both indices show a normal distribution (*p* > 0.2). When the groups are analyzed separately, there is a significant correlation for dyslexic children (R = 0.52, *p* = 0.03), but not for controls (R = 0.15, *p* = 0.57). This strongly suggests that, although not all dyslexics have a proprioceptive deficit, their qualitative levels of proprioception and reading are closely linked.

Tests carried out on a subgroup of 12 participants (6 dyslexics and 6 controls) showed that proprioception measured at both elbow and hip correlated with reading in dyslexics (Spearman R = 0.83, *p* = 0.04), but not in controls (Spearman R = 0.03, *p* = 0.96). This suggests that proprioceptive disorders in dyslexia are global.

The absence of elbow and hip involvement in reading contradicts the hypothesis that the sensory disorders observed in dyslexia result from a deficit in reading practice [7].

## 3. Postural Control and Dyslexia

Proprioception plays a fundamental role in motor learning, movement prediction and automation. Its most widely-explored function is to help maintain balance and adapt posture, with fluid, coordinated movements despite environmental and gravitational constraints [8]. Proprioceptive information collaborates with visual, vestibular and plantar information [9]. The analysis of posture is an essential indicator of the quality of a subject’s motor control, making it a subject of major interest in our study.

As early as 1973, Frank and Levinson demonstrated postural instability in dyslexic children, suggesting cerebellar and vestibular dysfunction [10]. This hypothesis was developed further in 2001 by Nicolson, who proposed that the reading deficit and sensorimotor symptoms result from a failure to automate cognitive and/or motor learning, linked to a slight dysfunction of the cerebellum [11]. The cerebellum, involved in the processing of proprioceptive information, plays a key role in these functions. Thus, although Nicolson did not explicitly mention it, his model is partly in line with the work of Henrique Martins da Cunha, who postulated a link between postural deficiency and dyslexia [5]. However, this hypothesis has not been widely accepted in the scientific community, particularly due to the variability in the methodologies used to assess motor and balance disorders [12,13]. Similarly, the magnocellular theory proposed by Stein—based on the hypothesis of an anatomical and functional alteration of the magnocellular system—remains debated [14]. This system plays a key role in several sensory modalities, notably visual, auditory and cutaneous [15,16]. There are anatomo-functional connections between proprioception and the magnocellular system, via structures such as the thalamus, parietal cortex and cerebellum. This multimodal integration is essential for dynamic perception of the body and environment, influencing postural control, motor skills and coordination [17].

First, in order to objectively assess their static balance abilities, we measured postural stability on a stabilometric platform in 50 dyslexic and 38 normo-reading children [18]. Tests included standardized monopodal and bipodal stances (feet open at 30°), with eyes open and closed. Data analysis focused on statokinesigram length, center of pressure fluctuations, and the weighted mean power frequency (MPF) on the lateral and anteroposterior axes.

**Figure 2:**
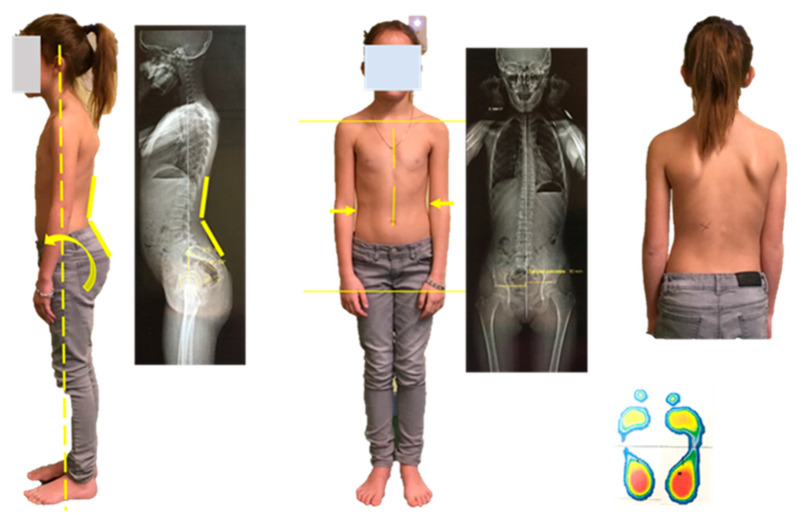
Postural characteristics of a dyslexic child include an anteroposterior imbalance of the center of pressure and a scoliotic attitude. In this case, the pelvis is projected forward; pelvis is higher on the left side (arrows); in others, it may be the head. At times, both the pelvis and head are projected anteriorly, creating a nearly normal appearance. Center of pressure recordings confirm this forward projection, despite podometric analysis indicating increased weight distribution on the heels.

The results show that over 50% of dyslexic children have impaired postural performance, with the exception of MPF on the lateral axis. The main differences were observed on the anteroposterior axis, corroborating clinical observations [19] (Figure 2).

This anteroposterior imbalance is usually associated with a scoliotic attitude, in line with manipulations of the genes encoding proprioceptive mechanoreceptor ion channel proteins (Piezo2, Asic2): they systematically induce scoliotic attitudes in mice [20,21]. Crucially, anteroposterior instability alters the function of the diaphragm, which must then behave not simply as a respiratory muscle, but also as a postural muscle [22].

In addition, dyslexic children showed marked difficulty in maintaining a unipodal stance, irrespective of which side was used. This difficulty is particularly marked when the eyes are closed, with 80% of failures on the right support and 60% on the left, revealing an increased dependence on visual information for motor control.

These initial results therefore suggest the presence of altered postural control in dyslexic children, particularly marked in the anteroposterior axis and under unipodal weight-bearing conditions. The involvement of the diaphragm as a postural muscle and the strong dependence on visual information to maintain balance are particularly noteworthy.

Secondly, in order to explore the link between impaired postural control and reading difficulties, we analyzed stabilometric parameters during a cognitive task [23]. At the same time, we evaluated the effect of the therapeutic protocol proposed by Martins da Cunha, combining prisms, foot orthoses and postural reprogramming exercises.

We compared the performance of three groups: twelve untreated dyslexic children, fifteen dyslexic children who had been receiving treatment for more than three months, and twelve non-dyslexic children. The children were assessed under two conditions: in the first, they simply had to maintain their balance by staring at a point in front of them; in the second, this task was coupled with silent reading of a Stroop test, which measures selective attention, cognitive control and inhibition of automatisms.

Analysis of center of pressure (CoP) displacements revealed a significant increase in mean CoP velocity during the reading task, but only in untreated dyslexic children, indicating increased postural instability under cognitive load. Conversely, an opposite trend was observed in children who had undergone treatment: For 13 out of 15 children, mean CoP speed decreased during reading. For 12 out of 15 children, the area of the 90% confidence ellipse was reduced, reflecting improved postural stability. The performance of treated children was thus close to that of non-dyslexic children

These results suggest that increased cognitive load can disrupt postural control in dyslexic children. However, adapted treatment promotes better sensorimotor integration, normalizing the interaction between cognitive demands and balance maintenance.

**Figure 3:**
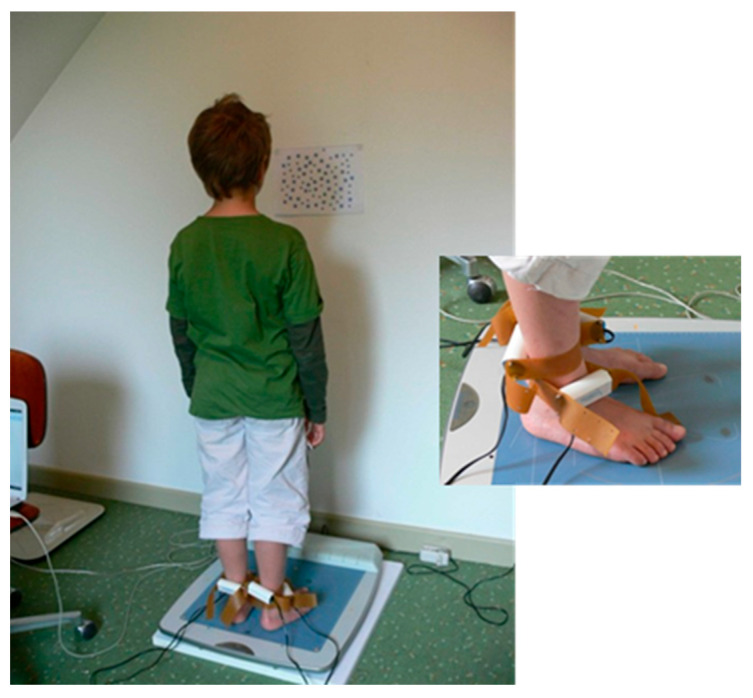
85 Hz vibrations delivered simultaneously to the Achilles tendons and tibialis anterior muscles of both legs to disrupt proprioceptive afferents while the child stares at an A4 sheet containing drawings of small and large stars without moving his head.

In a third phase of the study, we examined whether the integration of proprioceptive signals during balance control was naturally more impaired in dyslexic children, and whether this impairment became more pronounced under increased attentional load [24]. We also assessed whether the therapeutic protocol combining prisms, foot orthoses and postural reprogramming exercises mitigated these effects [25]. The study compared three groups of children: 30 untreated dyslexic children, 51 dyslexic children treated for three months, and 42 non-dyslexic children (control group). Participants were asked to maintain stable posture under two attentional conditions. In the first (control condition), they stared at a single point 40 cm in front of them. In the second (attention condition), they visually explored and counted, without moving their heads, an A4 sheet containing 40 large and 40 small stars evenly distributed. These two attentional conditions were crossed with two proprioceptive stimulation conditions. In the first, no vibrations were applied to the ankles. In the second, vibrations (85 Hz) were delivered simultaneously to the Achilles tendons and tibialis anterior muscles of both legs to disrupt proprioceptive afferents (Figure 3).

In treated dyslexic children, the prisms and foot orthoses were removed a few minutes before the test, to assess any persistence of treatment effects.

Analysis of the data revealed that the mean speed of CoP displacement under vibration conditions increased significantly more in dyslexic children, both treated and untreated, compared with the control group. This effect was independent of the attentional task.

However, in the absence of vibration, the attentional performance of the treated children was comparable to that of the control group, while that of the untreated dyslexic children remained significantly impaired.

These results suggest impaired proprioceptive integration in postural control, as well as impaired attentional abilities in dyslexic children. However, a significant improvement in attention to balance was observed after treatment. The alignment of the performance of treated children with that of normo-readers, even after the sensory treatment had been withdrawn, indicates a potential remanence of its effects, probably linked to neuronal reprogramming mechanisms.

## 4. Sleep Alterations in Dyslexic Children: Correlations with Neuroplasticity and Postural Control

Children’s sleep is structured in successive cycles alternating between slow-wave and REM sleep. Slow wave sleep is essential for the elimination of metabolites resulting from daytime neuronal functioning, and plays a key role in regulating attention. REM sleep, on the other hand, corresponds to a phase of increased cerebral plasticity, essential for neurogenesis in young children and, subsequently, for the automation of perceptual, cognitive and motor skills [26]. The functional and anatomical neuronal organization of the brain is thus highly dependent on the quality of REM sleep. The brain of a dyslexic child exhibits a disrupted neuronal organization [1].

Organically, REM sleep is characterized by generalized muscle hypotonia, including the thoracic respiratory muscles, leaving respiration mainly dependent on the diaphragm. This muscle is involved in postural control, particularly in stabilizing the lumbar spine [20,27,28]. In the supine position, dyslexic children are clinically observed to adopt a dominant thoracic breathing pattern, with difficulty in voluntarily executing purely diaphragmatic breathing. Thoraco-abdominal asynchronism is frequently observed, resulting in an inability to voluntarily dissociate thoracic and abdominal breathing.

Clinically, even in the absence of an associated attentional disorder or intense workload, dyslexic children and their families report persistent morning fatigue. This finding prompted an in-depth investigation of sleep in this population. It has already been shown that dyslexic children’s REM sleep duration is reduced, with a lengthening of onset latency, and that they have alterations in cycle organization correlated with the extent of dyslexia [29,30,31]. In typical readers, sleep plays a fundamental role in memory consolidation and learning new words. However, these benefits are not observed in dyslexic children [32,33].

Firstly, simple observation of the polysomnographic recordings of 18 dyslexic children without attentional disorder seen in consultation revealed a high percentage of inspiratory flow limitation with micro-arousals (Respiratory Effort-Related Arousal = 18.2 ± 7.9) associated with an Apnea Index per Hour (AHI) of 3.6 ± 3.5. These observations suggested the presence of upper airway resistance syndrome (SARVAS, or UARS) in this population [34]. On this basis, an exploratory study protocol (still in progress and whose results have not been published yet) was proposed to 21 dyslexic children aged 8 to 12, who also had no attentional disorder and were receiving speech therapy. The aim was to evaluate the impact of proprioceptive treatment, based on the use of calibrated prisms, proprioceptive insoles and abdominal breathing exercises at bedtime, on their sleep quality, attentional faculties and reading performance. Breathing exercises were self-learned from videos freely available on the internet, or used the Guillarme Method, the choice being left to the patient [35,36,37]. We also wanted to know whether 3D actimetry could be used for sleep monitoring as an alternative to polysomnography. Polysomnography remains the gold standard for sleep exploration, but access to it is difficult and costly.

Assessments were conducted at three distinct time points: three months prior to the proprioceptive examination (M-3), at the time of the examination (M0); and three months following the initiation of treatment (M+3), using a standardized protocol. Sleep quality was assessed using the Sleep Disturbance Scale for Children (SDSC), completed by parents after observing their child during a three-hour nighttime period [38]. Nocturnal motor activity was recorded using 3D actigraphy, which measured movements every 30 s over three consecutive school nights; this method was selected due to local limitations in access to polysomnography. Attentional performance was evaluated using the Test of Everyday Attention for Children (TEA-Ch) [39]. Reading abilities were assessed with a 180-s leximetry test (the Alouette test), which includes numerous linguistic challenges and is widely used in France to evaluate oral reading strategies [40]. This allowed for the calculation of three indices: accuracy ([correctly read words/total number of words] × 100), speed ([correctly read words/reading time] × 180), and efficiency (precision/speed × reading time).

Considering as abnormal only the symptoms occurring once or twice a week or more frequently (scores ≥ 3), the parental evaluation of the sleep questionnaire demonstrated a significant improvement in symptoms (*p* < 0.001) (Table 1).

Actimetry data showed no significant change in the sleep profile of dyslexic children after treatment. It is likely that the sampling frequency of the measurements did not allow for the detection of short-duration micro-awakenings that do not cause movements.

Assessment of attentional functions using the TEA-Ch revealed a significant improvement in attentional flexibility after treatment (*p* = 0.017). However, no significant improvement was observed for selective attention (*p* = 0.47) or sustained attention (*p* = 0.99).

The children showed a significant improvement in their reading performance on the leximetry test (Table 2).

The improvement in reading ability is even more marked when the results are compared between M-3 and M+3, suggesting that speech therapy alone, whose benefits are not significant between M-3 and M0, is considerably enhanced by the addition of proprioceptive treatment.

These results confirm that dyslexic children suffer from altered sleep patterns, which can be improved by proprioceptive therapy. The effectiveness of this approach may be explained by its ability to compensate for a detrimental diaphragmatic dysfunction. Indeed, in dyslexic children with proprioceptive disorders with postural deficiency, the diaphragm should not be considered solely as a respiratory muscle but also as a postural muscle. The impairment of its function in an upright position appears to persist in a supine position, leading to inspiratory difficulties and multiple micro-awakenings (Upper Airway Resistance Syndrome) during REM sleep. These observations suggest a potential link between REM sleep alterations, motor control abnormalities and the fMRI deficits characteristic of dyslexia, opening up new avenues of research into the interactions between sleep, brain plasticity and the development of reading skills [41,42].

The study must be completed by replacing 3D actigraphy with polysomnographic recordings, which remain the gold standard for reliably reflecting sleep quality.

## 5. Motor Imaging and Dyslexia

Motor imagery consists in mentally representing a movement without physically executing it [43,44]. Neuroimaging studies show that the same brain areas (such as the motor cortex, cerebellum and basal ganglia) are activated when a movement is imagined and when it is actually performed. The ability to represent action internally is important in the prediction, correction and procedural learning processes of motor skills. But it is also involved in language processing. Classically, anterior cortical regions, mainly motor, are considered to be involved in speech production, while posterior regions are involved in speech comprehension. However, recent research, inspired by theories of perception and action, shows that listening to language activates motor processes similar to those involved in production [45]. It would therefore be inaccurate to dissociate the processes involved in understanding speech from the motor mechanisms involved in its production. The motor system seems to play a particularly important role in discriminating speech signals in noisy environments [46]. This finding is in line with the frequent complaints of dyslexic people, who report hypersensitivity to noisy sound environments, particularly in school settings.

Despite these fundamental data on speech perception processes and their potential involvement in phonological disorders, the mental representation of action has not been studied in dyslexic children.

We therefore applied this cognitive technique to 18 dyslexic adolescents (with no associated signs of dyspraxia or attention deficit disorder) and 18 age-matched normo-readers [47]. They were asked to physically perform a visually-guided pointing task (rapid movement of a pencil tip in increasingly smaller squares) and to imagine the same task, indicating its imagined duration. Assessment of the mental representation of the action was based on two criteria: (1) compliance with Fitts’ law, according to which the time required to execute a linear movement increases in proportion to the difficulty of the task (i.e., pointing at smaller targets generates slower movements than pointing at larger targets), and (2) maintenance of isochrony between the durations of actual and imagined movements [48].

Our main findings showed that the dyslexic group performed both real and mental movements significantly more slowly than the control group. They also demonstrated deficits in action representation. This was evidenced by a lack of conformity to Fitts’ law in the mental condition and by the absence of isochrony between real and mental movement times.

Furthermore, in both groups, motor imagery accuracy correlated with combined word reading scores (fluency/accuracy) (regular, irregular and pseudowords). Note that this correlation was present for each word reading subtest, and particularly for tests exploring phonological abilities.

## 6. Spatial Attention and Dyslexia

The visuo-attentional theory of dyslexia suggests that a dysfunction in visual processing and visual attention is added to the phonological deficit [49]. In dyslexic children, this disorder is manifested by a reduction in the visual-attentional span, which corresponds to the number of letters they can process simultaneously in a single glance. This limitation, of which the child is unaware, led us to explore a possible alteration in physiological neglect in this population [50]. This investigation is all the more relevant as proprioceptive signals from the eye muscles influence both the deployment of attention and its mapping in visual space [51,52]. It has also been shown that pathological neglect disorders can be modulated by the use of prisms, as well as by proprioceptive modifications of the cervical muscles [53,54].

Understanding how dyslexic children perceive space may have practical implications for the design of their school environment. To explore these differences, we conducted a study of 10 right-handed children using the line bisection test [55]. This test, which involves marking the center of a line with a pen, is commonly used to assess spatial representation tendencies: a slight leftward shift is observed in healthy individuals whilst a rightward bias is observed in cases of spatial neglect [56].

The results showed that dyslexic children had an overall rightward bias compared with non-dyslexic children, while retaining their ability to analyze spatial contexts [57]. This idea of “reverse pseudoneglect” in dyslexia was reinforced by circle centering experiments [58]. In these tests, children had to re-center circles according to different sensory cues (visual and proprioceptive), with starting positions on the left or right.

We observe that:

- In the lateral dimension, their response was influenced by the hand with which they started the exercise in proprioceptive condition.
- In the radial dimension, they tended to project forward in the visuo-proprioceptive condition and to make more backward errors in the proprioceptive condition.
- Dyslexic children showed a forward bias when exploring clockwise, and better accuracy when exploring anti-clockwise, particularly when starting on the left.

To better understand how these particularities influence spatial organization in the school environment, we developed a new experiment: the landmark alignment task [59]. In this exercise, two parallel aluminum bars were placed radially, and the children had to position a landmark on one bar in order to align it with a reference landmark placed by the examiner on the other bar.

The results showed that dyslexic children had a forward bias on the left bar, suggesting either an under-representation of peripersonal space on the left, or an overestimation of space on the right. This radial forward bias was also correlated with reading delay, underlining the importance of taking better account of these particularities in the support of dyslexic children.

## 7. Interactions Between Postural Control, Proprioception and Visual Localization

Most of the eye movements used during reading are horizontal saccades. They enable fixations to be obtained which must be as stable as possible at the level of the words’ “center of gravity” in order to facilitate their decoding. These very precise movements require sensorimotor training and excellent visual spatial localization. The latter can be modified experimentally by proprioceptive modifications (vibrations) in ocular muscles, but also in muscles located at a distance, and by stimulation of the plantar surface [60,61]. In the adult good reader, many fixations are crossed, the axis of the left eye crossing that of the right eye. This horizontal disparity is accompanied by a vertical disparity, with a gap exceeding the width of a character in 33% of cases [62]. Even greater disparities are observed in typically developing children up to the age of 10 or 11 years old [63,64].

Saccades and fixations have very different characteristics in dyslexics [65]. They are accompanied by convergence disorders, horizontal heterophoria and fixation disparity [66]. The latter differs from that of children who are normal readers only during reading tasks [67]. Vertical fixation disparities in downward gaze have not been documented, as their measurement is technically unresolved.

**Figure 4:**
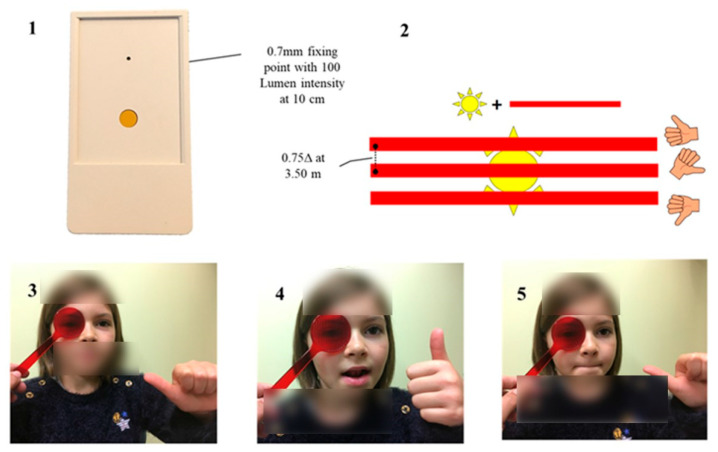
The vertical Maddox test is performed with a calibrated fixation point (1). To ensure that responses do not interfere with oral sensorimotricity, part of which is conveyed by the trigeminal nerve, as is ocular proprioceptive information, the child responds with his thumb to indicate whether the line is in, above or below the light (2). Here, the position of the red line is centered at the start of the test (3), then shifts upwards when V2 is stimulated, when the tip of the tongue comes into contact with the retro-incisor papillae (4), before returning to center (5) when VII is stimulated (contraction of the orbicularis of the lips).

The observation of particular postural control in dyslexic children led us to examine their binocular balance using the vertical Maddox test. A close relationship has been demonstrated between postural changes and the presence of vertical heterophoria [68,69]. Our analyses have highlighted a specific type of vertical microheterophoria, systematically found in dyslexic children, irrespective of the type of dyslexia [70] (Figure 4).

This heterophoria is described as labile because it can be modulated by sensory variations affecting distant sensors, notably in the oral area and on the plantar surface [71,72]. A precise protocol makes it possible to study the effect of different remote stimuli [70]. Each time a variation in vertical heterophoria is observed, the lability index, reflecting the instability of binocular vision, gains one point (Table 3).

Characterized by a very low amplitude (0.25 to 0.75Δ), this *labile* vertical heterophoria can only be detected with specific equipment, including a luminous fixation point <0.7 mm, with an intensity of 100 lumens at 10 cm, projecting a very fine red line behind the Maddox screen. Despite its low value, it cannot be compensated for by voluntary adjustments. What’s more, the use of low-power vertical prisms cannot stabilize the lability that characterizes it, unlike oblique prisms that reinforce the action of the upper oblique muscles.

**Figure 5:**
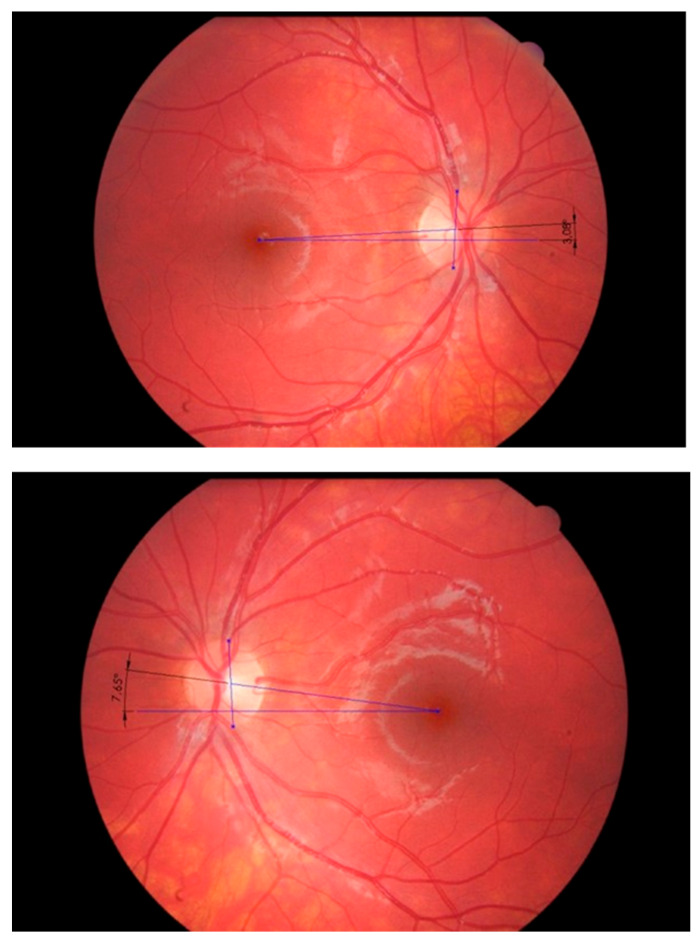
Measurement of torsion from retinography images shows asymmetry with frequent left excyclotorsion, indicating hypertonia of the inferior oblique muscle. The torsion is calculated from the angle between the lines passing through the fovea, one being horizontal and the other passing through the center of the optic disc.

This labile heterophoria is systematically associated with subjective and objective cyclotorsion, suggesting an imbalance in oblique muscle action, characterized by hypertonicity of the lower oblique muscles and functional limitation of the upper oblique muscles (Figure 5).

Balance between these muscles is essential to neutralize antagonistic torsional forces during downward gaze orientation, particularly during reading. Indeed, during inferior adduction movements, a vertical and horizontal displacement of 20 to 30° is physiologically accompanied by a torsion of 3 to 8° [73]. In this common visual reading posture, the upper oblique muscles act to neutralize the torsional effects induced by the lower rectus muscles. These torsional movements are not under voluntary motor control but depend on postural reflexes, thus reducing the influence of efference copy in favor of ocular and general proprioception.

However, the effect of oblique prisms may prove insufficient, requiring additional regulation by direct or distal proprioceptive stimulation, particularly at the podal or oral level [71,72].

Roll suggested that changes in proprioceptive information were sufficient to give the impression of a change in the spatial location of the fixed light, without the eye actually moving [60,61]. This hypothesis is supported by analysis of iris movements: when a child perceives a change in the height of the red line under proprioceptive stimulation, the objective displacement does not correspond to the reported perception in 58% of cases, compared with only 3% in normal readers [74].

Thus, labile vertical heterophoria would not only reflect actual eye displacement, but rather a central dysfunction of visual spatial localization, linked to impaired proprioception and associated sensory sensors.

## 8. Dyslexia, Proprioception and Multisensory Integration

Learning to read relies on automating the coupling between phonemes and graphemes, a process that mobilizes several sensory modalities. In this context, we have studied the influence of auditory stimuli on visual perception when binocular vision is impaired, using the vertical Maddox test in normo-reading and dyslexic children [75,76].

**Figure 6:**
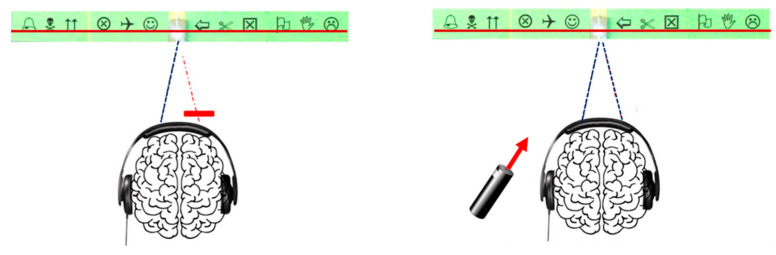
The presence of visual pseudo-scotomas is investigated by performing a vertical Maddox test (**left**) in front of each of the 2 eyes, or by preserving binocular vision (**right**) using a red laser beam. In both cases, vision is identical at the cerebral level, i.e., a red line aligned with a light, with drawings on either side of the light. Drawings allow the location of pseudo scotomas to be identified.

The experiment consisted in exposing the children to 45 biaural sound stimuli of 500 Hz and 1000 Hz, each lasting 1 s, in random order. During this exposure, they were asked to report any loss of visual perception and localize them using a line of drawings visible to the eye unmasked by the Maddox screen (Figure 6).

**Figure 7:**
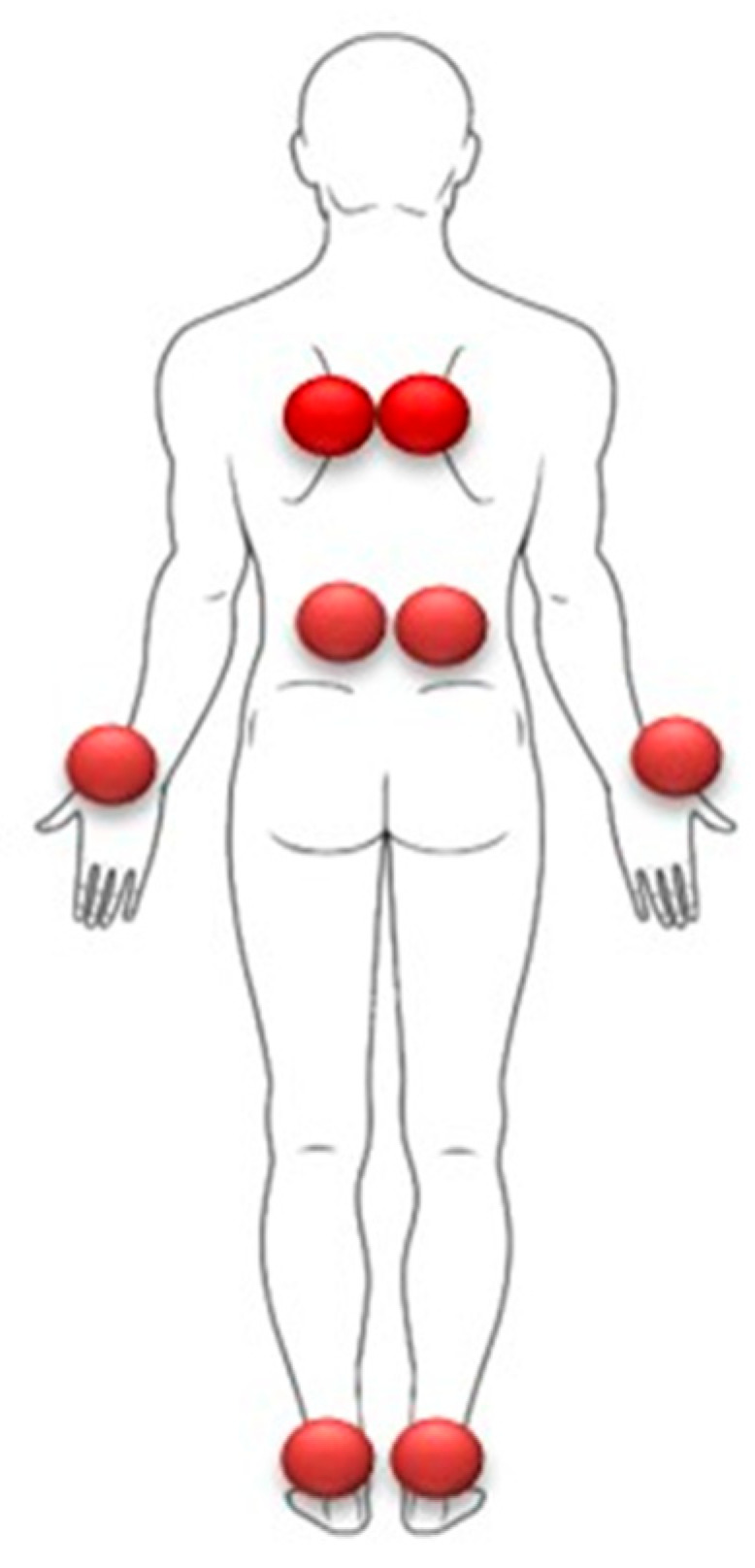
Direct proprioceptive stimulations are applied to muscles that play a role in postural regulation (paravertebral and ankle muscles) and those that do not (wrist muscles). The red circles corresponding to the proprioceptive stimulation zones.

We then extended this analysis by replacing the sound stimuli with proprioceptive changes induced by the application of muscle vibrations at 70 Hz for 2 s (Figure 7). To assess the specificity of postural proprioception in this phenomenon, the vibrations were applied to both postural muscles and muscles with no postural function (e.g., wrist). Furthermore, to determine whether the visual alterations were directly linked to a disturbance in binocular vision, we compared these results with those obtained by presenting an identical visual target using a red laser beam projected onto the light, this time in binocular vision.

**Figure 8:**
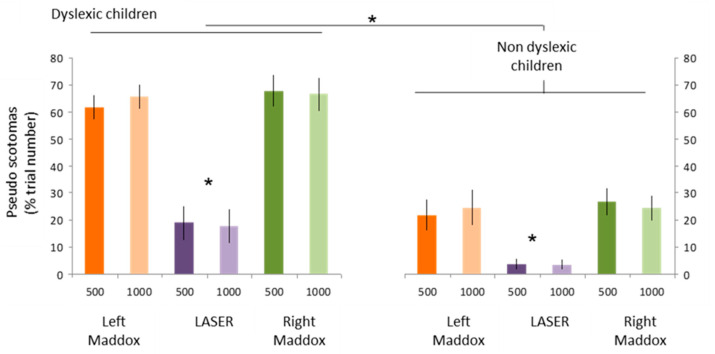
Frequency of occurrence of pseudo visual scotomas in dyslexic and non-dyslexic children during sound stimulation with modified binocular vision (Maddox left and right) or with preserved binocular vision (laser). (* for *p*-value < 0.05).

**Figure 9:**
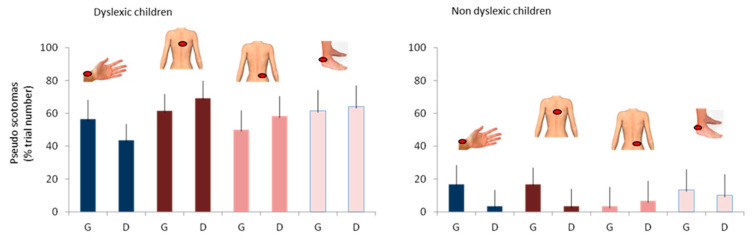
Frequency of occurrence of pseudo scotomas during the vertical Maddox test in dyslexic and non-dyslexic children—with direct proprioceptive stimulation on muscles that play a role in postural control or not. The red circles corresponding to the proprioceptive stimulation zones.

The results highlight several key elements:

(1) Visual loss caused by listening to sounds:
  - Auditory stimulation leads to visual perception disturbances in the form of visual losses called pseudoscotomas, as they do not correspond to organic but rather functional scotomas,
  - This phenomenon is not specific to dyslexic children, but it is clearly more marked in this population, (Figure 8),
  - Pseudo-scotomas only occur when binocular vision is disrupted by the Maddox screen.
(2) Temporal stability of pseudoscotomas: In the same subject, the same stimulation may or may not cause pseudoscotomas to appear.
(3) Spatial stability of pseudo-scotomes:
  - The same sound stimulus can induce visual alterations in different spatial regions, in a random and unpredictable way. This stochastic nature suggests that the brain, which relies on predictive mechanisms, has difficulty adapting.
(4) Effects of proprioceptive stimulation:
  - The application of muscular vibrations at 70 Hz induces effects similar to auditory stimulation. These visual disturbances appear independently of the postural function of the targeted muscles, suggesting that the phenomenon is based on a dysfunction of global proprioceptive processes, rather than a simple alteration of proprioception involved in postural regulation (Figure 9).

These results reinforce the hypothesis that dyslexia is associated with impaired multisensory integration, in which ocular and general proprioception play a central role, affecting visual perception when modulated by auditory or proprioceptive stimuli.

## 9. Statistical Analysis of Subjective Signs of Proprioceptive Dysfunction and Dyslexia

In dyslexic children, daytime proprioceptive dysfunction is therefore associated with postural disorders, as well as alterations in visual spatial perception and multisensory integration. These clinical manifestations could provide a complementary assessment element to the analysis of reading ability in children suspected of having dyslexia. Assessing sleep quality is also of major importance, given the fundamental role of proprioception in regulating diaphragmatic breathing during REM sleep. To assess the diagnostic value of these easily detected signs, we conducted a multicenter study of 103 dyslexic children and 110 normo-reader children aged 8 to 12. Conducted by 13 practitioners in 10 geographically distinct centers, it assessed the prevalence of 71 clinical signs, both daytime and nocturnal, frequently reported by dyslexic children [77]. These signs included those initially described by Henrique Martins da Cunha in the context of postural disorders, signs of sleep disturbance from the Sleep Disturbance Scale for Children, as well as elements collected during several thousand proprioceptive clinical examinations of dyslexic children [5,38].

Comparative analysis of subjective clinical manifestations between dyslexic and normo-reader children enabled a clear distinction to be made between the two groups. This differentiation led to the design of a 34-item questionnaire with a scoring system [78]. A total score above 80 was associated with a 21-fold increased risk of dyslexia, while a score between 35 and 44 corresponded to a 34-fold increased probability of belonging to the normo-reader group (Table 4).

This questionnaire is highly effective, provided parents take the time to observe their child’s sleep, could become a valuable tool for detecting the risk of dyslexia.

One of the notable findings of this study concerns the increased prevalence of nocturnal symptoms in dyslexic children.

In addition, a questionnaire concerning the sleep quality of the parents of the 103 dyslexic children showed that over 30% of these parents were undergoing treatment with nocturnal positive airway pressure for sleep apnea, despite their average age being only 43 years old. For comparison, data from Santé Publique France in 2022 indicated that 2.3% of the entire adult population in France was equipped with positive pressure devices for sleep apnea [79].

## 10. Management of Dysproprioception

The treatment of dysproprioception in dyslexics is based on a multimodal approach integrating three main therapeutic axes:

(1) Discreet deflection of light rays reaching the retina thanks to very low-power prisms (usually between 0.50 and 1.50Δ) designed to regulate the tone of the lower oblique muscles. The prisms are always bilateral, slightly asymmetrical in power, with a superior-external base, and are designed to correct vertical heterophoria and its lability. This optical modification leads to a global postural readjustment, affecting not only the ocular muscles, but also all proprioceptive chains down to the feet. Their very low power is necessary for their action on paravertebral muscle tone [80]. They do not induce habituation, and influence all proprioceptive chains, from the eye muscles to the lower limbs.
(2) Postural adaptation with specific insoles incorporating thin overlays (0.3 to 2 mm) to modulate plantar pressure sensors and act on vertical heterophoria [72]. The location of stimuli is codified and determined according to their postural repercussions [81]. These insoles adjust the perception of support on the ground, inducing targeted proprioceptive adaptation.
(3) Ergonomic optimization and respiratory reprogramming. Ergonomics during visual tasks are adapted to balance postural tone and minimize involvement of the upper oblique muscles. The use of a desk inclined at 30° promotes symmetrization of proprioceptive information. At the same time, specific daily breathing exercises aim to normalize diaphragmatic function, thereby improving sleep quality, particularly REM sleep. This optimization reduces micro-arousals, limiting their deleterious effects on attention and procedural memory [82]. Interaction between the ocular and oral proprioceptive systems via the trigeminal nerve can generate sensory interference. Additional management may be required in cases of persistent primary swallowing, oral ventilation, dental occlusion disorders or enlarged tonsils. These abnormalities can play a detrimental role in the severity of sleep disorders, by disrupting lingual and pharyngeal motricity and/or decreasing upper airway airflow [83].

## 11. Discussion

All these results reinforce the idea that dyslexia is not limited to a phonological deficit, but is accompanied by an ocular and global proprioceptive disorder for which the role of the ophthalmologist is crucial. To understand the link between these two disturbances, it is necessary to abandon the idea of a strict separation of cerebral functions. Indeed, modern theories of action and perception challenge this view and suggest that motor and perceptual circuits, especially those of speech, are closely interconnected [45,46].

Proprioceptive disorders must be considered both during wakefulness and sleep. Its nocturnal repercussions, marked more by upper airway resistance syndrome than by numerous apneas, could affect neurogenesis in early childhood and brain plasticity throughout life. These processes are essential to learning, whether it be reading or, more broadly, all sensorimotor and cognitive acquisitions. Disturbances in neuronal organization linked to deviant neurogenesis during REM sleep could also explain the diversity of neuronal anomalies observed on fMRI. Although this hypothesis still requires further research, it opens up new prospects in both exploratory and therapeutic terms.

Several studies have already highlighted the importance of assessing and addressing motor skills in children with dyslexia [84,85,86]. To date, it remains difficult to determine whether the motor disorders observed in these children are merely comorbid symptoms or whether they play a direct role in the etiology of the reading disorder, as proposed by the cerebellar and magnocellular hypotheses [11,14]. Within this theoretical framework, a dysfunction of the proprioceptive system may be implicated. This dysfunction could underlie the daytime impairments previously described and may play a central role in the disruption of diaphragmatic function, thereby compromising sleep quality. Such disturbances could contribute to a global delay in neural maturation, accompanied by diverse motor and sensory deficits.

Thus, an intervention aimed at restoring optimal sensory conditions during wakefulness and correcting alterations in procedural memory during REM sleep may represent a key step, complementing conventional rehabilitative interventions. A recent randomized controlled study, carried out in another laboratory, examined in great detail the effects of proprioceptive intervention by analyzing eye movements during silent reading in dyslexic children [87]. They were randomly assigned to either normal speech therapy or a proprioceptive and speech therapy intervention, in which they received both the usual speech therapy and a proprioceptive intervention. Silent reading performance and eye movements were measured before and after the intervention (after nine months). Reading performance improved only in the proprioceptive treatment group. The analysis of eye movements showed that the performance of children who received proprioceptive treatment became identical to that of normal readers in terms of oculomotor processes (saccade size), cognitive processes of word recognition (lexical access), and reading automation processes. Also of note was the observation of an effect on the reading of little-used words in the French language, suggesting a positive action on phonological decoding.

## 12. Conclusions

Our work is in line with the scientific consensus establishing the existence of phonological disorders in dyslexia. However, based on the interactions between perception and action, they suggest that these disorders should be considered as being intertwined with alterations in sensorimotor control.

Our results suggest a possible redefinition of dyslexia, which would no longer be considered as an isolated disorder, but rather as a symptom among other varied manifestations. These could be linked to ocular and general proprioceptive dysfunction, but also to a sleep pathology of proprioceptive origin, responsible for neurodevelopmental abnormalities during infancy.

Exploring the role of proprioception in dyslexia calls for a reassessment of current diagnostic methods, as screening professionals do not always have the skills required to identify proprioceptive dysfunctions. An interdisciplinary approach and closer collaboration between specialists are therefore essential if we are to refine our understanding of dyslexia, optimize its management, and undertake joint research that goes beyond the simple presence of the reading disorder.

## Figures and Tables

**Table 1:** Scores of the Sleep Disturbances Scale for Children [30].

	M-3/M0 *p*-Value	M0/M+3 *p*-Value
1. Number of hours of sleep	0.16	0.08
2. Time to fall asleep	0.84	0.03
3. The child reluctantly goes to bed.	1	0.09
4. Child has trouble falling asleep at night.	0.81	0.04
5. The child is anxious or frightened when it’s time to go to sleep.	0.81	0.07
6. The child jerks or shakes certain parts of his body as he falls asleep.	0.38	<0.001
7. The child makes repetitive gestures such as rocking or banging his head as he falls asleep.	0.13	0.13
8. The child experiences vivid dream scenes while falling asleep.	0.01	<0.001
9. The child sweats excessively when falling asleep.	0.35	0.003
10. The child wakes up more than twice a night.	0.46	0.20
11. After waking up during the night, the child has trouble getting back to sleep.	0.07	0.20
12. The child has frequent leg spasms or twitches during sleep, or often changes position during the night.	0.84	<0.001
13. The child has difficulty breathing at night.	0.26	0.04
14. Child stops breathing at night.	0.16	0.31
15. The child snores.	1	0.28
16. The child sweats excessively at night.	0.50	0.003
17. You have observed that the child is sleepwalking.	1	0.03
18. You have observed that the child talks in his sleep.	0.41	0.01
19. The child grinds his teeth in his sleep.	1	0.02
20. The child wakes up screaming or so confused that you can’t get him to talk, but he has no memory of these events the next morning.	0.38	0.002
21. The child has nightmares that he can’t remember the next morning.	0.86	0.09
22. The child is difficult to wake up in the morning and this seems unusual for a child.	0.08	0.12
23. The child wakes up in the morning feeling tired.	0.46	0.008
24. The child feels unable to move when he wakes up in the morning.	1	0.04
25. The child is easily sleepy during the day.	0.08	1
26. The child suddenly falls asleep in inappropriate situations (in the car, when quiet, or in the middle of noise, etc.).	0.65	0.02
27. The child has an abnormal head position while sleeping (head tilted back and in extension).	0.02	0.001
28. The child salivates a lot at night or there are traces of drool on the pillow in the morning.	0.23	<0.001
29. Child complains of a headache in the morning.	0.72	0.03
30. The child breathes with its mouth open while sleeping.	0.87	<0.001
31. The child still pees or often gets up at night to go to the toilet.	0.42	0.003
32. The child has trouble remembering lessons learned the night before (even though he knew them the night before).	0.815	<0.001
33. The child tends to be a little sleepy at times at school.	0.107	0.14
*p*-value of score differences (counting only signs with a frequency ≥ 3	0.69	<0.001

For each symptom, after carefully observing their child’s sleep for at least 4 h, at M-3, M0, and M+3, parents were asked to assign a score based on the frequency of occurrence: 1 = Never; 2 = Occasionally (once or twice a month or less); 3 = Sometimes (once or twice a week); 4 = Often (three to five times a week); 5 = Every day. The M-3/M0 column represents the difference (*p*-value) in progression between the questionnaire completed without treatment three months before the clinical examination (M-3) and at the time of the clinical examination (M0). The M0/M3 column represents the progression after three months of proprioceptive treatment. Note that only scores of 3 or higher were considered abnormal and were used for the comparison of periods.

**Table 2:** Evolution of reading performance for 3 months before the examination (M-3/M0) and after 3 months of proprioceptive treatment (M0/M+3). During both periods, the children received speech therapy. The evaluation between M-3 and M+3 is very positive and superior to that of M0/M+3, suggesting that the effect of speech therapy is significantly enhanced by proprioceptive treatment.

	M-3/M0	M0/M+3	M-3/M+3	*p* M-3/M0	*p* M0/M+3	*p* M-3/M+3
Number of words correctly read	10.14	14.31	25.90	0.036	0.003	<0.001
Accuracy	1.23	4.11	5.39	0.763	0.014	0.002
Speed	13.29	14.45	29.66	0.025	0.004	<0.001
Efficiency	134.80	122.99	107.87	0.103	0.016	<0.001

**Table 3:** Example of a Maddox test to explore the effect of remote stimulation on response lability. UP Arrow = red line above the light, Down Arrow = line below the light, OV = line centered on the light. The lability index increases by one point each time the response differs from the previous situation, whether for one eye or both eyes.

		Maddox in Front of Right Eye	Maddox in Front of Left Eye	Changes in the Lability Index
1	Seated in natural position without plantar support.	OV	↓	
2	Sitting upright without plantar support (modification of spinal proprioception)	OV	OV	+1
3	Bielchowski test on the right shoulder	more ↓		+1
4	Bielchowski test on the left shoulder		more ↑	+1
5	Tip of tongue pressed against retro incisor papillae (V2 stimulation)	OV	↓	+1
6	Tip of tongue pressed against lower incisors (stimulation of V3)	↓	↓	+1
7	Tight lips (VII stimulation)	OV	OV	+1
8	Standing upright with foot support on hard ground	OV	↓	+1
9	Upright standing with plantar support on foam insoles (modification of plantar exteroception)	OV	↓	0
Lability Index				Σ = 7

For example, if the response in situation 2 differs from that in situation 1 for the left eye only, this corresponds to one point of lability. Similarly, if the response in situation 5 differs from that in situation 4 for both eyes, this still accounts for only one point. The Bielschowsky test, which identifies the hypertonic oblique muscle in each eye, is considered separately, as it tests one eye at a time. Each change observed during this test also adds one point to the lability index. Compared with the previous situation, the responses on the 2 eyes have changed 5 times and 2 times with the Bielchowski test (hypertonia of the inferior oblique for the right eye and hypertonia of the superior oblique for the left eye), giving a lability index of 7. When the patient straightens up or stimulates the VII, the 2 eyes are in vertical orthophoria. These 2 stimulations are considered corrective. This result gives indications on the general treatment plan.

**Table 4:** Questionnaire for calculating the risk of dyslexia. A score above 80 multiplies this risk by 21.

**FOR EACH SYMPTOM, INDICATE THE NUMBER CORRESPONDING TO THE FREQUENCY** 1 = Never, 2 = Occasionally (1 or 2 times/month), 3 = Sometimes (1 to 2 times/week), 4 = Often (3 to 5 times a week), 5 = Every day.	
**15 Questions for PARENTS (after examining the child’s sleep for at least 3–4 h of continuous sleep)**	**SCORE**
The child jerks or moves body parts when falling asleep.	
The child has agitated daydream scenes when falling asleep.	
The child moves his legs a lot when he sleeps or often changes position during the night or kicks the bed covers.	
You have observed that your child sleepwalks	
Your child has nightmares (terrors) that he can’t remember the next morning.	
He has great difficulty waking up in the morning	
The child feels unable to move around, feels very tired when waking up in the morning.	
The child is sleepy during the day (falls asleep easily in the car, quiet, …)	
The child salivates a lot at night or there are traces of drool on the pillow in the morning.	
Child complains of a headache in the morning	
The child breathes with its mouth open while sleeping	
The child still pees or often gets up at night to go to the toilet.	
The child has trouble remembering lessons learned the night before (even though he knew them the night before).	
The child tends to be a little sleepy at times at school	
The child has an abnormal head position while sleeping (head tilted back and in extension).	
**19 Questions to be answered by the CHILD**	
**Muscular dimension**	
Do you feel tired even if you haven’t exerted yourself physically or mentally?	
Is it hard for you to stand by and do nothing?	
Do you get a headache after school?	
Do you have recurring lower or upper back pain?	
Do your legs ever hurt?	
Is it hard for you to stare at a text (or a person) up close?	
Do you ever see double when you’re tired, after reading a text?	
Do you get out of breath quickly when you exert yourself (for example, as soon as you run)?	
Can you see blurred up close after reading a few lines (with your glasses, if you have them)?	
**Spatial dimension**	
Is it difficult for you to walk on something narrow (a beam, for example)?	
Is it difficult for you to catch an object on the first try—a ball, for example?	
Do you fall easily or twist your ankles?	
You bite your tongue or cheeks when you eat.	
Do you bump into simple obstacles (doorframes for example, …) as if you didn’t perceive the space around you properly?	
**Perceptive dimension**	
Do you feel like you’re reading without understanding what you’re reading?	
Do you find it hard to concentrate for long?	
When someone speaks to you, do you feel like you don’t really understand what you’re hearing?	
When you read, you have the impression that you can’t see properly: you skip words, you miss line breaks, …	
It’s hard for you to express an idea when you’re talking, and you have trouble constructing your sentences properly?	
**TOTAL SCORE—Enter the SUM of the scores for all 34 questions**	

## Data Availability

The data presented in this review are available on request from the corresponding author (except for data published in journals that impose copyright).
